# Influence of Pain and Discomfort in Stroke Patients on Coping Strategies and Changes in Behavior and Lifestyle

**DOI:** 10.3390/brainsci11060804

**Published:** 2021-06-17

**Authors:** Silvia Reverté-Villarroya, Rosa Suñer-Soler, Sílvia Font-Mayolas, Antonio Dávalos Errando, Esther Sauras-Colón, Andrea Gras-Navarro, Mireia Adell-Lleixà, Georgina Casanova-Garrigós, Elsa Gil-Mateu, Marta Berenguer-Poblet

**Affiliations:** 1 Department of Nursing, University Rovira Virgili, Tarragona, 43500 Tortosa, Spain; silvia.reverte@urv.cat (S.R.-V.); andrea.gras@urv.cat (A.G.-N.); mireya.adell@urv.cat (M.A.-L.); georgina.casanova@urv.cat (G.C.-G.); elsa.gil@urv.cat (E.G.-M.); marta.berenguer@urv.cat (M.B.-P.); 2 Hospital de Tortosa Verge de la Cinta, Pere Virgili Institut, 43500 Tortosa, Spain; esther.sauras@iispv.cat; 3 Health and Health Care Research Group, Department of Nursing, University of Girona, 17003 Girona, Spain; 4 Quality of Life Institute, University of Girona, 17004 Girona, Spain; silvia.font@udg.edu; 5 RETICS Research Group, Department of Neurosciences, Hospital Universitari Germans Trias i Pujol, 08916 Badalona, Spain; adavalos.germanstrias@gencat.cat

**Keywords:** pain, coping, quality of life, stroke, adherence, health promotion, behaviors

## Abstract

The implementation of prevention strategies can reduce the risk of having a stroke. This prospective, longitudinal, multicenter observational study of 82 patients describes health habits, quality of life, coping strategies, and physical and neurological status at 3 months and 1 year after stroke. The EuroQoL-5D quality of life scale (EQ-5D) and the coping strategy measurement scale (COPE-28) were used to assess pain and discomfort, and behavioral and lifestyle changes. Significant differences were observed in the pain or discomfort levels of those patients with behavioral and lifestyle changes. Correlation was also found between pain or discomfort and the coping strategies associated with active emotional support at 1 year after stroke. The results of the pain or discomfort dimension were not, however, associated with better adherence to treatment. Pain and discomfort could have a predictive value in changes in lifestyles and behaviors but not for treatment adherence in patients who have had a stroke, which is significant at 1 year. In addition to important active coping strategies such as social support, these changes in behavior and lifestyle following a stroke are long-term and should therefore be assessed during the initial examination.

## 1. Introduction

Stroke incidence and mortality and the risk of recurrence have decreased in the last few decades [1]. This is largely due to advances made in acute-phase treatments and the management of risk factors. Prevention strategies, such as secondary prevention management of stroke risk factors, can determine a cumulative reduction of up to 80% in the relative risk of vascular events [1]. These prevention strategies involve making lifestyle changes, for example, interventions for behavioral changes, and promote therapeutic adherence [2].

Even though evidence supporting certain interventions for implementing behavioral changes in stroke patients is increasing, results have not improved [3]. Many strategies for achieving behavioral and/or lifestyle changes are based on Prochaska’s model of change [4], although its evidence is controversial due to limitations when involving more than one intervention [5]. Cognitive–behavioral strategies are also used to achieve behavioral changes, but long-term studies are still needed to demonstrate their benefits [2].

Proposed interventions with stroke patients include case management, reminders, a therapeutic plan and checklist, educational materials, and audits of the impact on therapeutic adherence [6]. Consequently, low therapeutic adherence, along with difficulty in adopting behavioral changes and a healthy lifestyle, leads to the poor management of risk factors, low self-efficacy, physical–cognitive deficits, low quality of life, and an increased possibility of experiencing a recurring vascular event [7].

Recently, psychosocial factors have been incorporated as potentially modifiable stroke risk factors [3]. Stress is a triggering factor that impacts psychological well-being [8,9], including during the post-stroke phase.

Coping strategies are among the most effective adaptive mechanisms against the effects of chronic disease, such as a stroke. Theorists Lazarus and Folkman argued that although stress affects each individual’s physiology differently, the dynamic process they described as “coping” incorporates these variable features over time [10,11]. Thus, depending on the coping capacity of each patient, they are more or less able to select the most effective coping strategy for their stressor, in this case, the disease [12].

Few studies in the literature relate coping strategies, quality of life, behavioral changes, and therapeutic adherence after stroke in a well-defined sample, including long-term follow-up. The present study intends to shed light on a topic of interest and general concern about a global health problem that is poorly studied. Thus, the primary objective of this study was to describe the variables of health habits (therapeutic adherence and behavioral changes), quality of life, coping strategies, and physical and neurological status at 3 months and at 1 year after stroke. The secondary objective was to draft a model to predict health-habit changes at 1 year.

## 2. Materials and Methods

### 2.1. Study Design

A prospective, longitudinal, multicenter observational study was carried out in two mid-size medical centers in Spain that participated in the Endovascular Revascularization With Solitaire Device Versus Best Medical Therapy in Anterior Circulation Stroke Within 8 Hours (REVASCAT) clinical trial [13]. Patients who had met the REVASCAT inclusion criteria (ClinicalTrials.gov, accessed on 8 June 2021, Identifier: NCT01692379) were recruited between March 2013 and March 2016 and followed up until January 2017. During this period, 82 of the 206 subjects from REVASCAT were included in this sub-study. These were ischemic-stroke patients treated with thrombectomy and coping (ICTO) and treated for an acute cerebral vascular accident at the Hospital Universitari Germans Trias i Pujol (HUGTiP) and the Hospital Universitari Vall d’Hebrón (HUVH), two of the four recruitment centers.

### 2.2. Patients

**Figure 1:**
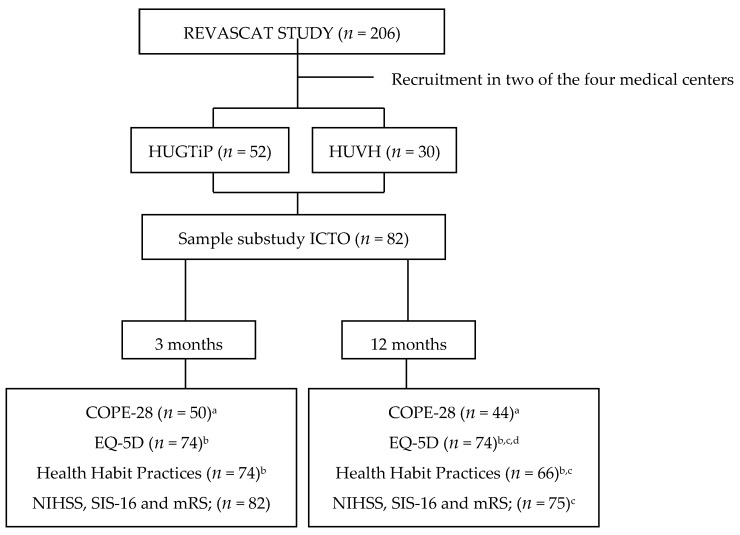
Flowchart showing the recruitment and assessment process for subjects in sub-study ICTO. Reasons for not being evaluated: ^a^: aphasia/dysphasia; ^b^: global aphasia; ^c^: death; ^d^: cognitive deterioration. HUGTiP: Hospital Universitari Germans Trias i Pujol; HUVH: Hospital Universitari Vall d’Hebrón.

The inclusion criteria were as follows: patients between 18 and 85 years old with occlusion in the proximal anterior circulation treated with reperfusion therapies within 8 h of symptom onset; pre-stroke functional ability between 0 and 1 on the modified Rankin scale (mRS) (ranging from 0 (no symptoms) to 6 (death)) [14]; and a baseline score of at least 6 points on the National Institutes of Health Stroke Scale (NIHSS) [15], which ranges from 0 to 42, with higher values indicating more severe deficit. The main exclusion criteria were imaging tests showing a large ischemic core, as indicated by an Alberta Stroke Program Early Computed Tomography Score (ASPECTS) of more than 8 on computed tomography (CT) without the use of contrast material or a score of less than 6 on diffusion-weighted magnetic resonance imaging (MRI). ASPECTS values range from 0 to 10, with higher values indicating less of an infarct burden [13]. Details regarding recruitment and eligibility are provided in Figure 1.

### 2.3. Data Collection

Several sociodemographic details of the patients participating in the study were collected, including age, gender, marital status (married, single, divorced or separated, and widower), education level (no studies, primary, secondary, vocational training, and university), employment status before the stroke (active, unemployed, and retired), baseline family context (alone, with family and/or caregiver, and center or residence), and substance use (alcohol and smoking). Clinical characteristics, such as etiology, topography, and laterality of stroke, were also collected.

Patients’ neurological features were described through the NIHSS and the Stroke Impact Scale-16 (SIS-16), which ranges from 40 to 80, with higher values indicating better patient condition. The NIHSS was used to quantify stroke-related neurologic deficit, and the SIS-16 to assess physical functions in patients at 3 months and at 1 year after stroke. In addition, stroke topography and patient morbidity were analyzed to study the origin of the pain.

The primary purpose of our study was to assess the use of coping strategies in response to stress according to the Spanish version of the Brief COPE (COPE-28) and its relationship with the pain or discomfort dimension [16,17,18]. This instrument consists of 28 items that evaluate 14 different dimensions: active coping, planning, positive reframing, acceptance, humor, religion, use of emotional support, use of instrumental support, self-distraction, denial, venting, substance use (alcohol or drugs), behavioral disengagement, and self-blame. The scale includes several questions with possible answers ranging from 0 (“I didn’t do this at all”) to 3 (“I did this a lot”), including intermediate scores. The higher the score achieved in each dimension, the greater the coping strategies used. In terms of psychometric properties, Cronbach’s alpha for this evaluation was calculated at 3 months (α = 0.75) and 1 year (α = 0.68). Results of the Brief COPE should be interpreted with caution, as the instrument assumes that individuals can accurately define their coping styles.

The second aim was to evaluate the results of the EQ-5D scale, which quantifies quality of life [18]. This questionnaire has two components: health status description and the evaluation (which uses a visual analogue scale). However, we only focused on the first part for this study, which measures generic health status in terms of five dimensions (mobility, self-care, daily activities, pain or discomfort, and anxiety or depression). Our results are mainly focused on the pain or discomfort dimension. Regarding psychometric properties, Cronbach’s alpha for these items was calculated at 3 months (α = 0.891) and 1 year (α = 0.838).

**Figure 2:**
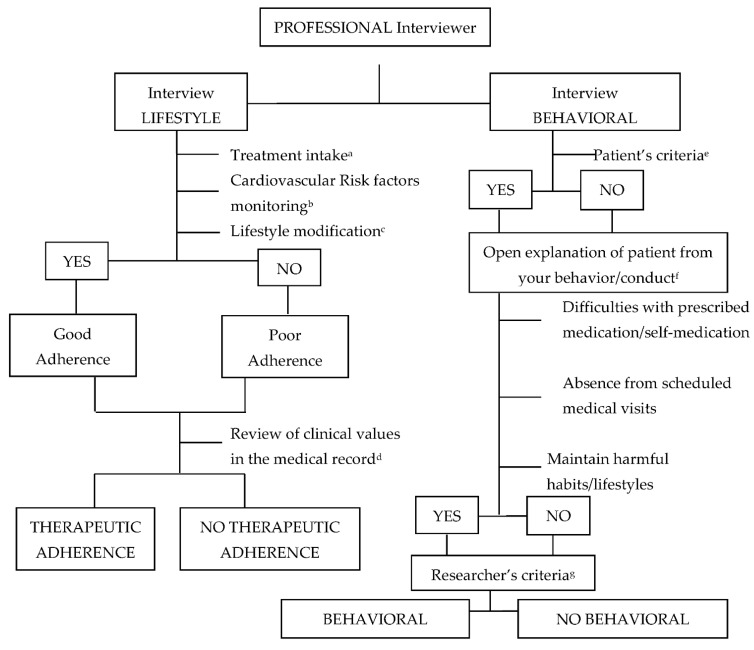
Algorithm to classify lifestyle and behavioral changes. ^a^ Patient self-report on taking prescribed medication without missing any doses [19]. ^b^ Cardiovascular risk factors present and review of the control measures adopted by the patient [19]. ^c^ Changes in lifestyle, such as a healthy diet and aerobic exercise [23]. ^d^ Investigator check on the information provided by the patient through their medical history. ^e^ Self-report by the patient on whether they adopted a healthy behavior change. ^f^ In both cases, the patient was openly interviewed to verify whether or not behavior had changed through the following: presence of difficulties in taking the prescribed medication and whether the patient was self-medicating; absence from scheduled visits by patient’s doctor or nurse and maintaining harmful habits/lifestyles. ^g^ According to the results of the interview with the patient, a change in behavior was considered or not at the discretion of the researcher.

The third aim was to assess health habit practices through therapeutic adherence and behavioral changes at 3 months and 1 year after stroke. An algorithm was created to determine whether or not patients had adhered to treatment and if they had adopted any behavioral changes at 3 months and 1 year after the stroke. The algorithm was developed on the basis of the 2016 European Guidelines on cardiovascular disease prevention in clinical practice and WHO recommendations [19,20]. Two interviews designed to assess different aspects of lifestyle and behavioral changes were conducted (Figure 2). Figure 2 summarizes the interviewer’s assessment of adherence to treatment and adoption of a healthier lifestyle depending on their compliance with items such as attending appointments, following a healthy diet [21], and avoiding substance use (no alcohol consumption and no smoking). Four variables were identified as a result: (1) therapeutic adherence at 3 months, (2) therapeutic adherence at 1 year, (3) behavioral changes at 3 months, and (4) behavioral changes at 1 year. Each of these variables could take one of two values, defined in yes or no terms: yes, there was therapeutic adherence; no, there was no therapeutic adherence; yes, there were behavioral changes; no, there were no behavioral changes. For instance, occasionally missing a dose would result in an assessment of non-adherence. The interviews were conducted face to face at the external neurology clinics of the participating hospitals during patients’ follow-up visits and as part of the usual appointments for these patients.

### 2.4. Analysis

Descriptive statistics were applied to summarize patients’ sociodemographic and clinical characteristics and to analyze several variables. The chi-square test was used to analyze differences between groups for both dichotomous and qualitative variables, and Spearman’s correlation coefficient was used to study the correlation between quantitative variables at 3 months and 1 year after stroke. In addition, two logistic regression models were carried out to explain the lifestyle and behavioral changes at 1 year after stroke. Therefore, seven variables were selected (age, gender, pain or discomfort, emotional support, COPE-28 total score, NIHSS, and SIS-16) to identify the best explanatory model. Statistical significance was established at a *p* value <0.05 and 95% confidence interval (CI). All analyses were performed using IBM SPSS^®^ Statistics software v.26.

### 2.5. Ethical Considerations

The study was approved by the ethics committees of the involved hospitals. The ID protocol of the study is ICTO/2014. All procedures were performed in accordance with the principles of the 1964 Helsinki Declaration and its later amendments [22]. All participants involved in the study provided written informed consent.

## 3. Results

Patient sociodemographic and clinical characteristics are shown in Table 1. The median age of the participants in the study was 69 years, and 52.4% were men. Most participants were married (75.6%), and the level of education varied, with a higher proportion having only primary studies (31.7%) or vocational training (24.4%). Moreover, 74.4% of patients had retired before the stroke, and a high percentage of the participants lived with their family and/or caregiver (84.1%). Most of them declared moderate alcohol consumption (79.3%), and 64.6% were not smoking. The etiology of stroke was mainly cardioembolic (69.5%), and according to its topography, the predominant type of stroke was total anterior circulation infarct (TACI) (86.6%). Regarding laterality, 45.1% of participants had a left hemisphere stroke, while 54.9% had a right hemisphere stroke.

Changes in lifestyle were assessed using the algorithm shown in Figure 2 to classify therapeutic adherence and behavioral changes (Table 2). At 3 months, the percentage of patients that had made lifestyle (67.5%) or behavioral (57.1%) changes was higher than the percentage of participants that had not (32.5% and 42.9%, respectively). At 1 year after stroke, the percentages remained similar. With regard to neurological features, the median NIHSS score decreased one point from the score at 3 months to the score at 1 year after stroke (4 vs. 3); the median SIS-16 score, on the other hand, increased during this period (53 vs. 61).

In order to determine what caused lifestyle and behavioral changes in patients, differences in each of the EQ-5D dimensions (mobility, self-care, daily activities, pain or discomfort, and anxiety or depression) were studied between the groups of patients that had undergone these changes and those that had not. While statistically significant differences were not found in any of the EQ-5D dimensions at 3 months (data not shown), at 1 year, significant differences were observed in the pain or discomfort dimension of patients with behavioral changes (*p* = 0.005), and with lifestyle changes (*p* = 0.051) (Table 3). There were no patients in the group with no lifestyle or behavioral changes with much pain or discomfort. The topography of stroke, its laterality, and the morbidity of the patients were all not associated with the pain or discomfort dimension.

We assessed the correlation between the pain or discomfort dimension considered as a score and the different dimensions of the COPE-28 scale, and we found statistically significant correlation between pain or discomfort and emotional support at 1 year after stroke with Spearman’s correlation coefficient of 0.282 (*p* = 0.047, Table 4). Instrumental support was borderline, with a Spearman’s correlation coefficient of 0.272 (*p* = 0.056, Table 4). However, there were no statistically significant differences at 3 months. This suggests that the greater the pain or discomfort after havinga stroke, the higher the long-term need for instrumental and emotional support (consolation or understanding) from other people.

Lastly, logistic regression models were performed to explain the lifestyle and behavioral changes at 1 year after stroke, the time point at which more interesting results were found. In both cases, the analyzed variables in the model were age, gender, the pain or discomfort dimension of the EQ-5D scale, emotional support, total COPE-28 score, and neurological status according to NIHSS and SIS-16. The behavioral change model equation included two statistically significant variables, namely, the pain or discomfort dimension and the total COPE-28 score (OR = 4.646, *p* = 0.042; OR = 0.922, *p* = 0.035, respectively; Table 5). According to our previous results, pain or discomfort was a trigger for long-term behavioral changes. Nevertheless, none of the variables mentioned above were statistically significant in the equation of the lifestyle change model (data not shown); therefore, these variables could not predict therapeutic adherence.

## 4. Discussion

The primary purpose of this longitudinal study was to describe the relationship between coping strategies, quality of life, health habit practices (therapeutic adherence and behavioral changes), and physical and neurological status at 3 months and at 1 year poststroke. As coping strategies are usually analyzed in a stressful environment, such as a stroke, these strategies may vary depending on the influence of additional stressors [24]. Therefore, we can identify two general categories: coping strategies based on the problem and those based on emotion [16]. The longitudinal design of this study allowed us to conduct a simultaneous evaluation of the temporal stability of multiple psychological factors in a homogeneous cohort of stroke patients. Stroke patients who experienced more pain and discomfort seemed to need higher instrumental and emotional support at 1 year after stroke. This strategy is part of active coping, and it conditions emotional responses. Ensuring emotional support is a good strategy for coping with stressors, and it is associated with decreased symptoms of depression because it helps to improve the perception of psychosocial well-being [25]. In addition, emotional support is directly related to adaptive strategies for disease confrontation such as self-efficacy [26]. Pain and discomfort directly affect instrumental support, which tends to increase in the longterm (1 year after stroke). This problem-focused coping strategy is also associated with emotional support [27], and according to Morelli (2015), instrumental support strategies have a positive effect on the patients’ well-being only when accompanied by emotional support [28]. These findings highlight the benefits of using problem-focused coping strategies in a stressful context, especially for older adults.

Stroke patients show the need to relinquish control of their situation to others in the long term (>6 months), which evidences skillful use of coping strategies in the short term. Ramazanu (2020) highlights that patients’ partners or caregivers are the focus of attention in these cases, concluding that the patients’ reliance on active coping mechanisms and the participation of their spousal caregivers in social activities reduced stress levels when compared to patients who used other strategies [29]. Moreover, the neurological functions of the participants progressed favorably, showing fewer consequences 1 year after the stroke, which is associated with a better quality of life in the longterm [30,31]. In any case, it is necessary to strengthen effective strategies, such as planning, active coping, and seeking instrumental and emotional support, while changing those that are not useful, such as venting, denial, or self-blame [32].

The most relevant finding of our study is that behavioral and lifestyle changes in patients at 1 year after an ischemic stroke are conditioned by the presence of pain and discomfort. Patients rarely or never perceive the benefits of adopting healthy lifestyles or therapeutic adherence; even if they do, it is usually not for long [32,33,34,35]. Therefore, it is important to consider all aspects in this process from a biopsychosocial perspective and to outline strategies to improve patients’ quality of life, both in the short and the longterm, following a stroke [36]. Thus, our data suggest that having pain or discomfort could significantly influence behavioral and lifestyle changes at 1 year after the stroke.

Many stroke patients, especially those with ischemic stroke, report immediate and late-onset pain. Such pain can be caused by multiple mechanisms related to the patient’s neurological and functional status (musculoskeletal impairments and spasticity) following a stroke [37,38]. Surprisingly, however, identifying and treating poststroke pain is often not considered to be a priority within a comprehensive approach, resulting in less encouragement to continue psychological therapies when compared to physical rehabilitation [39]. Adequate pain management could improve patients’ mood, comfort levels, recovery, and quality of life [40,41].

Lastly, the secondary aim was to draft a model to predict health habit changes at 1 year after stroke. After this period, coping strategies of patients experiencing pain or discomfort were observed to impact the adoption of healthier habits.

Consequently, the evaluation and control of pain or discomfort in stroke patients should be a priority, as they condition patient evolution and the adoption of lifestyle and behavioral changes. Likewise, active coping strategies for the patient, the caregiver, and others involved should be incorporated into routine clinical practice.

### Limitations

This study used a longitudinal descriptive design. Given the predictive value of its variables, further longitudinal interventional studies are needed to corroborate the direction of the results. Even though our results are in the same direction as those of the scant literature on this subject, the logistic regression model explains the low variability of behavioral changes at 1 year after stroke through the analysis of pain or discomfort and coping strategies. Nonetheless, the results of the logistic regression model should be considered with caution, given that the sample size of this study was small, and missing patients could have introduced a bias that should also be listed as a limitation. Moreover, the numbers of cases did not have sufficient statistical power to find positive correlation with the 14 different dimensions of the Brief COPE scale. Therapeutic adherence and behavioral changes are complex processes, and this study followed a simple design without psychometric analysis for therapeutic adherence and the behavioral change algorithm. Therefore, this study favored non-adherence to treatment given the complexity of post-stroke patients.

## 5. Conclusions

Pain and discomfort have a predictive value in changes in lifestyles and behavior but not for treatment adherence in patients who have had a stroke, this being significant at 1 year. Moreover, for these patients, pain management requires active coping strategies, such as emotional support and understanding of the caregivers and others involved, who can also suffer the consequences of the stressor, i.e., the stroke. Taking this into consideration will positively impact patients’ recovery, well-being, quality of life, adoption of a healthy lifestyle, and better therapeutic adherence. Finally, this study contributes relevant findings to the scant literature on the subject.

## Figures and Tables

**Table 1:** Sociodemographic and clinicalcharacteristics of patients.

Sociodemographic Characteristics	Total (*n* = 82)
Age (years) [IQR]	69 [60.0–77.0]
Gender (male) (%)	43 (52.4)
Marital status (%)	
Married	62 (75.6)
Single	7 (8.5)
Divorced or separated	4 (4.9)
Widower	9 (11)
Level of studies (%)	
No studies	14 (17.1)
Primary	26 (31.7)
Secondary	13 (15.8)
Vocational training	20 (24.4)
University	9 (11)
Employment status before stroke (%)	
Active	16 (19.5)
Unemployed	5 (6.1)
Retired	61 (74.4)
Baseline family context (%)	
Alone	12 (14.7)
With family and/or caregiver	69 (84.1)
Center or residence	1 (1.2)
Alcohol/smoking habits (%)	
Alcohol intake	
Never	65 (79.3)
Moderate (≤280 g/week)	13 (15.9)
Severe (>280 g/week)	3 (3.7)
Former	1 (1.2)
Smoking	
Never	53 (64.6)
Moderate (10–20 cigarettes/day)	11 (13.4)
Excessive (>20 cigarettes/day) ^1^	5 (6.1)
Former	13 (15.9)
Etiology of stroke (%)	
Atherothrombotic	12 (14.6)
Cardioembolic	57 (69.5)
Unusual cause	1 (1.2)
Undetermined	12 (14.6)
Topography of stroke (%)	
PACI	11 (13.4)
TACI	71 (86.6)
Laterality of stroke (%)	
Left	37 (45.1)
Right	45 (54.9)

Age is represented with median [IQR], while the remaining variables are represented with number of cases (percentage). ^1^ data applied to men.

**Table 2:** Healthy lifestyles in patients through therapeutic adherence, behavioral changes, NIHSS, and SIS-16 scores at 3 months and 1 year after stroke.

	3 Months (*n* = 74)	1 Year (*n* = 66)	*p*-Value
**Lifestyle changes**			
Therapeutic adherence	52 (67.5)	46 (69.7)	0.549
No therapeutic adherence	25 (32.5)	20 (30.3)	
**Behavioral changes**			
Yes	44 (57.1)	38 (57.6)	1.000
No	33 (42.9)	28 (42.4)	
	**3 Months (*n* = 82)**	**1 Year (*n* = 75)**	
NIHSS score	4 [1.0–11.0]	3 [1.0–7.25]	0.412
SIS-16 score	53 [20.50–73.0]	61 [45.50–76.25]	0.274

Lifestyle and behavioral changes are represented with the number of cases (percentage), while NIHSS and SIS-16 scores are described with median value [IQR].

**Table 3:** Differences in the pain/discomfort dimension of EQ-5D between patients with and without behavioral and lifestyle changes at 3 months and 1 year after stroke.

	3 Months (*n* = 74)			1 Year (*n* = 66)		
Pain/Discomfort	Behavioral Changes	No Changes	*p*-Value	Behavioral Changes	No Changes	*p*-Value
No pain/discomfort	20 (47.6)	12 (36.4)	0.599	12 (31.6)	16 (57.1)	0.005
Moderate pain/discomfort	16 (38.1)	16 (48.5)		15 (39.5)	12 (42.9)	
A lot of pain/discomfort	6 (14.3)	5 (15.2)		11 (28.9)	0 (0.0)	
**Pain/Discomfort**	**Behavioral Changes**	**No Changes**	***p*-Value**	**Behavioral Changes**	**No Changes**	***p*-Value**
No pain/discomfort	21 (42.0)	11 (44.0)	0.899	17 (37.0)	11 (55.0)	0.051
Moderate pain/discomfort	21 (42.0)	11 (44.0)		18 (39.1)	9 (45.0)	
A lot of pain/discomfort	8 (16.0)	3 (12.0)		11 (23.9)	0 (0.0)	

Variables are shown in number of cases (percentage), and chi-squared test is used to assess differences between groups.

**Table 4:** Correlation between each of the COPE-28 items and the pain/discomfort dimension of the EQ-5D scale considered as a score at 3 months and at 1 year after stroke.

	Active Coping	Planning	Emotional Support	Instrumental Support	Religion	Positive Reframing	Acceptance
**3 months: (*n* = 50)**							
Spearman’s ρ	0.057	−0.024	0.152	0.108	−0.040	−0.056	−0.069
*p*-value	0.693	0.869	0.291	0.457	0.783	0.697	0.633
**1 year: (** ***n* = 44)**							
Spearman’s ρ	0.225	0.242	0.282	0.272	−0.012	0.072	−0.096
*p*-value	0.117	0.090	0.047	0.056	0.935	0.621	0.508
	**Denial**	**Humor**	**Self-Distraction**	**Self-Blame**	**Behavioral** **Disengagement**	**Venting**	**Use of** **Substances** **^1^**
**3 months: (*n* = 50)**							
Spearman’s ρ	−0.172	0.057	0.052	0.126	−0.025	0.084	−0.142
*p*-value	0.233	0.694	0.717	0.385	0.864	0.562	0.325
**1 year: (*n* = 44)**							
Spearman’s ρ	0.163	−0.149	0.106	0.009	0.094	−0.019	0.275
*p*-value	0.259	0.302	0.466	0.948	0.518	0.894	0.053

^1^ Alcohol or drugs.

**Table 5:** Logistic regression model to explain behavioral changes at 1 year after stroke.

Variable	B	Standard Error	OR	(CI 95%)		*p*-Value
				Lower Limit	Upper Limit	
Constant	−8.439	6.117	0.000			0.168
Age	0.018	0.036	1.018	0.949	1.093	0.614
Gender (male)	0.624	0.681	1.867	0.492	7.089	0.359
Pain/discomfort (EQ-5D)	1.536	0.755	4.646	1.058	20.393	0.042
Emotional support	0.202	0.218	1.223	0.798	1.876	0.356
COPE-28 total score	−0.081	0.039	0.922	0.855	0.994	0.035
NIHSS score	0.397	0.274	1.487	0.869	2.543	0.148
SIS-16 score	0.088	0.054	1.092	0.983	1.213	0.101

Adjusted R^2^ = 0.305.

## Data Availability

The datasets collected during the current study are not publicly available due to the fact that the participants were not informed of the possibility that their data would be openly available.
